# HEARING LOSS AND FATIGUE IN MIDDLE-AGED AND OLDER ADULTS

**DOI:** 10.1093/geroni/igad104.0530

**Published:** 2023-12-21

**Authors:** Kening Jiang, Adam Spira, Frank Lin, Jennifer Deal, Nicholas Reed

**Affiliations:** Johns Hopkins Bloomberg School of Public Health, Baltimore, Maryland, United States; Johns Hopkins Bloomberg School of Public Health, Baltimore, Maryland, United States; Johns Hopkins, Baltimore, Maryland, United States; Johns Hopkins University, Baltimore, Maryland, United States; Johns Hopkins Bloomberg School of Public Health, Baltimore, Maryland, United States

## Abstract

Fatigue is associated with worse cognitive and physical functioning, psychosocial wellbeing, and quality of life. Hearing loss may be a risk factor for fatigue, perhaps through greater cognitive demand to compensate for degraded auditory signal. However, prior studies are predominantly in audiology clinical samples, which may be oversampled for individuals experiencing hearing-loss-related fatigue. We conducted a cross-sectional analysis including 3,031 participants from the National Health and Nutrition Examination Survey (NHANES) 2015-18. Fatigue was based on self-reported frequency of feeling tired or having little energy over the past 2 weeks. Hearing loss was defined as better-ear 4-frequency (0.5, 1, 2, 4 kilohertz) pure-tone average >25 decibels hearing level. Multivariable-adjusted multinomial logistic regression was used to model the relative risk ratios (RRR) of reporting a higher vs. reference frequency category of fatigue associated with hearing loss, adjusting for age, sex, race/ethnicity, education, smoking, drinking, occupational and off-work noise exposure, body mass index, comorbidities, and depressive symptoms. In this nationally representative sample of middle-aged and older adults (40-80 years, 48% male, 10% Black), 713 (24%) had hearing loss. When compared to normal-hearing participants, hearing-loss participants were more likely to report fatigue for more than half the days (RRR=2.16, 95% confidence interval [CI]: 1.24, 3.75) and nearly every day (RRR=2.09, 95% CI: 1.24, 3.52) relative to not feeling fatigue at all. Hearing loss is associated with fatigue at the population level. Public health efforts to manage hearing loss might improve fatigue and associated downstream health outcomes.

